# Exposure to Lipopolysaccharide and/or Unconjugated Bilirubin Impair the Integrity and Function of Brain Microvascular Endothelial Cells

**DOI:** 10.1371/journal.pone.0035919

**Published:** 2012-05-07

**Authors:** Filipa L. Cardoso, Ágnes Kittel, Szilvia Veszelka, Inês Palmela, Andrea Tóth, Dora Brites, Mária A. Deli, Maria A. Brito

**Affiliations:** 1 Research Institute for Medicines and Pharmaceutical Sciences, Faculty of Pharmacy, University of Lisbon, Lisbon, Portugal; 2 Institute of Experimental Medicine, Hungarian Academy of Sciences, Budapest, Hungary; 3 Laboratory of Molecular Neurobiology, Institute of Biophysics, Biological Research Centre, Hungarian Academy of Sciences, Szeged, Hungary; 4 Department of Biochemistry and Human Biology, Faculty of Pharmacy, University of Lisbon, Lisbon, Portugal; Semmelweis University, Hungary

## Abstract

**Background:**

Sepsis and jaundice are common conditions in newborns that can lead to brain damage. Though lipopolysaccharide (LPS) is known to alter the integrity of the blood-brain barrier (BBB), little is known on the effects of unconjugated bilirubin (UCB) and even less on the joint effects of UCB and LPS on brain microvascular endothelial cells (BMEC).

**Methodology/Principal Findings:**

Monolayers of primary rat BMEC were treated with 1 µg/ml LPS and/or 50 µM UCB, in the presence of 100 µM human serum albumin, for 4 or 24 h. Co-cultures of BMEC with astroglial cells, a more complex BBB model, were used in selected experiments. LPS led to apoptosis and UCB induced both apoptotic and necrotic-like cell death. LPS and UCB led to inhibition of P-glycoprotein and activation of matrix metalloproteinases-2 and -9 in mono-cultures. Transmission electron microscopy evidenced apoptotic bodies, as well as damaged mitochondria and rough endoplasmic reticulum in BMEC by either insult. Shorter cell contacts and increased caveolae-like invaginations were noticeable in LPS-treated cells and loss of intercellular junctions was observed upon treatment with UCB. Both compounds triggered impairment of endothelial permeability and transendothelial electrical resistance both in mono- and co-cultures. The functional changes were confirmed by alterations in immunostaining for junctional proteins β-catenin, ZO-1 and claudin-5. Enlargement of intercellular spaces, and redistribution of junctional proteins were found in BMEC after exposure to LPS and UCB.

**Conclusions:**

LPS and/or UCB exert direct toxic effects on BMEC, with distinct temporal profiles and mechanisms of action. Therefore, the impairment of brain endothelial integrity upon exposure to these neurotoxins may favor their access to the brain, thus increasing the risk of injury and requiring adequate clinical management of sepsis and jaundice in the neonatal period.

## Introduction

The blood-brain barrier (BBB) is a dynamic interface between blood and brain compartments that protects nerve tissue from insults. Brain microvascular endothelial cells (BMEC), possessing unique properties, are considered the main constituents of the barrier. They regulate the selective passage of substances through the expression of specific influx and efflux transport systems [1]. ATP-binding cassette (ABC) transporters, such as the efflux transporter P-glycoprotein (P-gp), export potentially toxic compounds. A relevant transcellular vesicular transport mechanism at the BBB occurs through caveolae, which are dynamic pieces of membrane enriched in cholesterol and sphingolipids, as well as in the structural protein caveolin-1 [1]. Additionally, BMEC display cohesive intercellular junctional complexes, composed of tight junctions (TJs) and adherens junctions (AJs). TJs are formed by transmembrane proteins like claudins, occludin, tricellulin, junctional adhesion molecules, and cytoplasmic proteins, like the *zonula occludens* (ZO) family [2]. TJs are responsible for high transendothelial electrical resistance (TEER) and low paracellular permeability at the BBB [3], [4]. AJs are constituted by the transmembrane proteins vascular endothelial cadherins, nectins, platelet-endothelial cell adhesion molecule, and by the cytoplasmic catenins, comprising β-catenin [5]. BMEC, pericytes and astrocytes share a thick basement membrane that is composed of various extracellular matrix (ECM) classes of molecules [1]. Matrix metalloproteinases (MPPs) are known to digest basement membrane proteins and impair TJs integrity [1]. Pathological conditions affecting the integrity of intercellular junctions, BBB transporters or the basement membrane impair the barrier function of the BBB, which can lead to or further increase brain damage.

Sepsis reflects an uncontrolled systemic inflammatory response to an infection that can cause organ dysfunction, eventually leading to shock or even death [6]. Lipopolysaccharide (LPS) is the major component of the outer membrane of Gram-negative bacteria. It may circulate in low levels in the blood in certain diseases [7], but high levels suggest infection or sepsis. Rat BMEC have been shown to express the Toll-like receptors (TLR) 2, 3, 4, 6 and the membrane cluster of differentiation 14 (CD14), which binds LPS [8], [9]. When activated, these receptors trigger the release of pro-inflammatory cytokines into the brain parenchyma and induce neuroinflammation. Our previous studies have shown that binding of LPS to rat primary BMEC co-cultured with astrocytes leads to increased permeability, reduced TEER, alterations in intercellular junctions assembly, as well as to inhibition of P-gp activity [10]. These changes in BBB integrity may favor the access of neurotoxins as well as of microbial pathogens to the brain [7].

Unconjugated bilirubin (UCB), the principal end product of heme catabolism, circulates in the plasma almost entirely bound to albumin due to its low solubility in aqueous medium, and the concentrations of unbound (free) bilirubin are in the nM range [11]. At low or slightly elevated concentrations as those reported for Gilbert patients, who present up to 100 µM total serum bilirubin and a UCB to albumin molar ratio of 0.2, UCB is a powerful antioxidant, able to provide protection against cardiovascular diseases and cancer [12]–[14]. It was also shown that UCB formed by upregulation of heme oxygenase-2, which is constitutive and highly concentrated in neurons, protects these nerve cells from H_2_O_2_-induced loss of cell viability. However, at higher concentrations, UCB is no longer beneficial but instead induces neuronal death [15]. This dual behavior was observed in our own laboratory, where 10 nM free UCB were shown to protect neurons from H_2_O_2_-induced neuronal death, nuclear factor (NF)-kB activation and tumor necrosis factor (TNF)-α secretion, whereas 100 nM was neurotoxic [16]. These observations support the notion that UCB acts as a double-edged sword, either beneficial at low concentrations, or detrimental at elevated levels. Accordingly, in the experimental conditions that we have been consistently using with different cell types [17]–[19], which reproduce a moderate or severe hyperbilirubinemia [UCB to human serum albumin (HSA) molar ratios of 0.5 and 1.0, respectively], toxicity is observed and oxidative stress appears as a relevant event [20]–[22]. Hyperbilirubinemia is particularly common in the neonatal period due to the shorter life span of fetal erythrocytes, together with the immaturity of the hepatic clearance system [12]. These high levels of UCB become neurotoxic by inducing oxidative and nitrosative stress, mainly in neurons [23], [24], and the release of pro-inflammatory cytokines by astrocytes [25] and microglia [26]. Our most recent studies revealed that human BMEC exposed to UCB are also under nitrosative stress, as indicated by the upregulation of endothelial nitric oxide synthase and production of nitrites, the end-product of nitric oxide, and release cytokines, such as the vascular endothelial growth factor and interleukin (IL)-6 [27]. Interestingly, both nitric oxide and these cytokines are known to increase BBB permeability [28], [29]. Despite such indication of a hyperpermeability of the BBB upon exposure to UCB, direct evidence of the disruption of the barrier properties by UCB exposure has never been provided.

Hyperbilirubinemia and sepsis frequently occur as associated pathological conditions in newborn infants. Sepsis is considered as a neurotoxicity risk factor during neonatal hyperbilirubinemia [30] and a recent prospective study indicated that jaundiced infants with encephalopathy were more likely to have a coexistent infection than hyperbilirubinemic infants without brain damage [31]. Our previous studies showed that the inflammatory response and cell death in astrocytes are exacerbated by LPS [17], [32]. However, the effects produced by the combination of these compounds have never been studied in BMEC.

The aim of this study was to reveal the effects of LPS and UCB, alone or in combination, on the morphological and functional characteristics of rat BMEC in conditions mimicking sepsis and moderate to severe hyperbilirubinemia.

## Results

### LPS, UCB and their Combination Induce Cell Death in Rat BMEC

**Figure 1:**
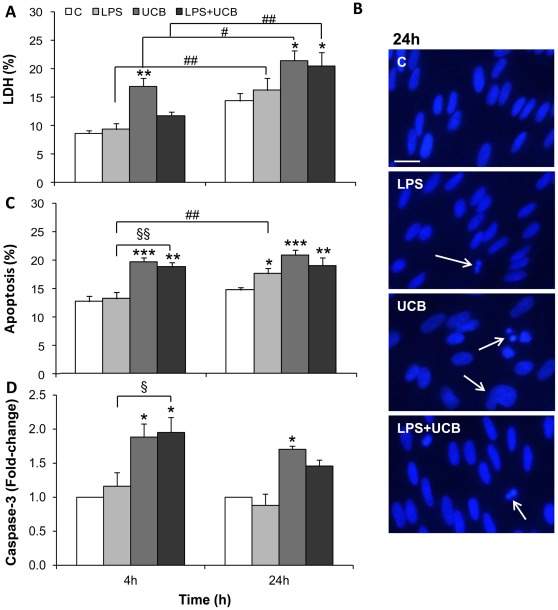
Lipopolysaccharide (LPS) and unconjugated bilirubin (UCB) induce cell death in brain microvascular endothelial cells. Culture medium was collected for determination of lactate dehydrogenase (LDH) activity (A). Nuclei were stained with Hoechst 33258 dye and morphological features of apoptosis are pointed (arrows) (B). The number of apoptotic nuclei was counted and results were expressed as percentage of the total number of nuclei (C). Cell lysates were obtained for caspase-3 activity determination (D). Results are mean ± S.E.M. from at least five independent experiments performed in duplicate. Scale bar, 20 µm. *P<0.05, **P<0.01 and ***P<0.001 *vs.* respective control; ^§^ *P<*0.05, ^§§^ *P<*0.01 *vs.* LPS at the same time-point; ^#^ *P*<0.05 and ^##^ *P*<0.01 from 4 h.

Primary cultures of rat BMEC are commonly used as simplified *in vitro* models of the BBB (for review see [29], [33]). In order to evaluate the impairment of rat BMEC viability by exposure to LPS and UCB, we assessed necrosis-like cell death by determining the release of intracellular lactate dehydrogenase (LDH) into the incubation medium. As seen in Fig. 1A, LPS triggered a mild, though not statistically significant, elevation of LDH release, which attained maximum levels of 9.4% and 16.2% at 4 and 24 h, respectively, corresponding to an overall time-dependent elevation (*P<*0.01) in the extent of cell death. With UCB, higher values were achieved (16.9% at 4 h and 21.4% at 24 h, *P<*0.01 and *P<*0.05, respectively, as compared to the respective control), reflecting a time-dependent loss of cell viability (*P*<0.05). There was no aggravation in necrosis-like cell death when cells were exposed to both LPS and UCB (LDH release at 24 h: 20.3%, *P<*0.05 as compared to the respective control), even though it similarly increased with the time of exposure (*P<*0.01).

Either UCB or LPS induced apoptosis in BMEC, as observed by staining endothelial nuclei with Hoechst 33258 dye followed by analysis of nuclear morphological features (Figs. 1B,C), in accordance with previous demonstrations [27], [34]. LPS induced apoptotic cell death in a time-dependent manner (*P<*0.01), reaching 18.0% (*P<*0.05) after 24 h treatment. When exposed to UCB, the percentage of cells with apoptotic morphology was already significantly increased at 4 h (19.6%, *P<*0.001) and sustained at 24 h (20.7%, *P<*0.001). As for LDH, there was no increment in UCB-induced apoptotic cell death by co-incubation with LPS. In contrast, apoptosis raised from 13.1% in LPS-treated cells to 18.7% in cells treated with both LPS and UCB for 4 h (*P<*0.01 as compared to LPS alone). To note that this difference disappeared as the incubation was extended to 24 h and death continued to increase in cells treated with LPS alone.

We also studied the activity of caspase-3 (Fig. 1 D). LPS induced no significant alterations in caspase-3 activity at the time-points studied, reaching a maximum elevation of 1.2-fold at 4 h. On the other hand, cells exposed to UCB had a significant increase in caspase-3 activity at the earliest time-point, attaining 1.9- and 2.0-folds for UCB and UCB with LPS, respectively (*P*<0.05). At 24 h cells treated with UCB alone revealed a sustained caspase-3 activity (1.7-fold, *P*<0.05).

### Activity of ABC Transporter P-gp is Impaired by LPS and UCB

**Figure 2:**
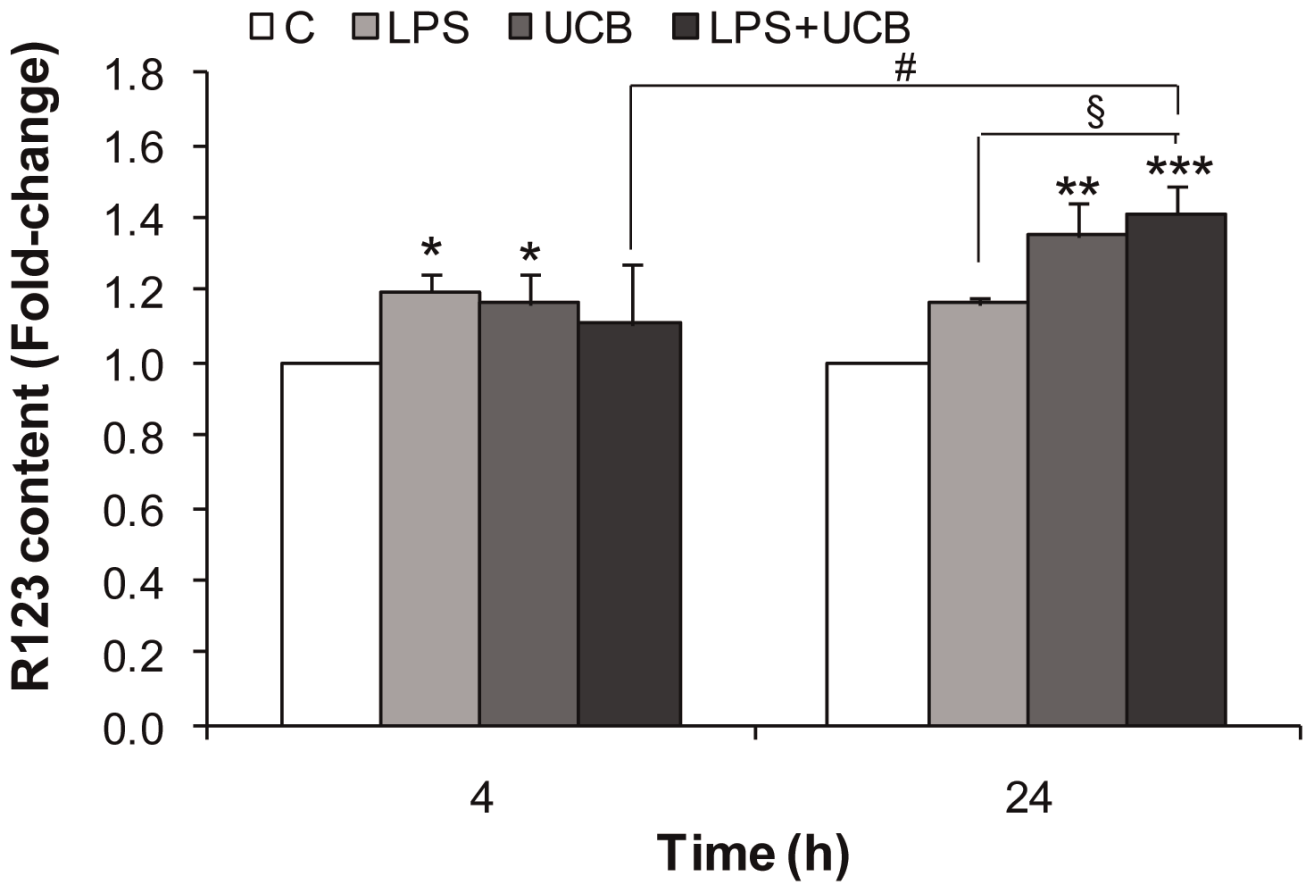
Lipopolysacharide (LPS) and unconjugated bilirubin (UCB) inhibit P-glycoprotein (P-gp) activity in brain microvascular endothelial cells. To evaluate if P-gp is functioning properly, it was measured the protein’s ability to remove the substrate rhodamine 123 (R123). After treatment, cells were incubated for 30 min with 10 µM R123. Total protein extracts were collected, R123 accumulation fluorescent emission was determined and results were expressed per mg of protein. Values presented are means ± S.E.M., from at least four independent experiments performed in duplicate. *P<0.05, **P<0.01 and ***P<0.001 *vs.* respective control; ^§^ *P<*0.05 *vs.* LPS at the same time-point;^ #^ *P*<0.05 from 4 h.

We studied the effects of LPS and UCB on the efflux transporter P-gp activity (Fig. 2), based on the measurement of cellular accumulation of rhodamine 123 (R123), the main substrate dye used to assess P-gp function. Accumulation of the substrate reflects a decreased activity of the efflux transporter, as have been recognized and used, particularly in primary cultures of BMEC [10], [35]. Results were expressed as fold-change from control, which presented ∼114 ng R123/mg protein. P-gp activity was already diminished following 4 h exposure to LPS or UCB, as indicated by the 1.2-fold elevation in intracellular levels of R123 (*P<*0.05). Exposure to both LPS and UCB increased P-gp inhibition with time (*P<*0.05) and aggravated the inhibition induced by LPS alone at 24 h (1.2-fold for LPS *vs.* 1.4-fold for LPS plus UCB, *P<*0.05).

### Activities of MMP-2 and MMP-9 are Increased by LPS and UCB

**Figure 3:**
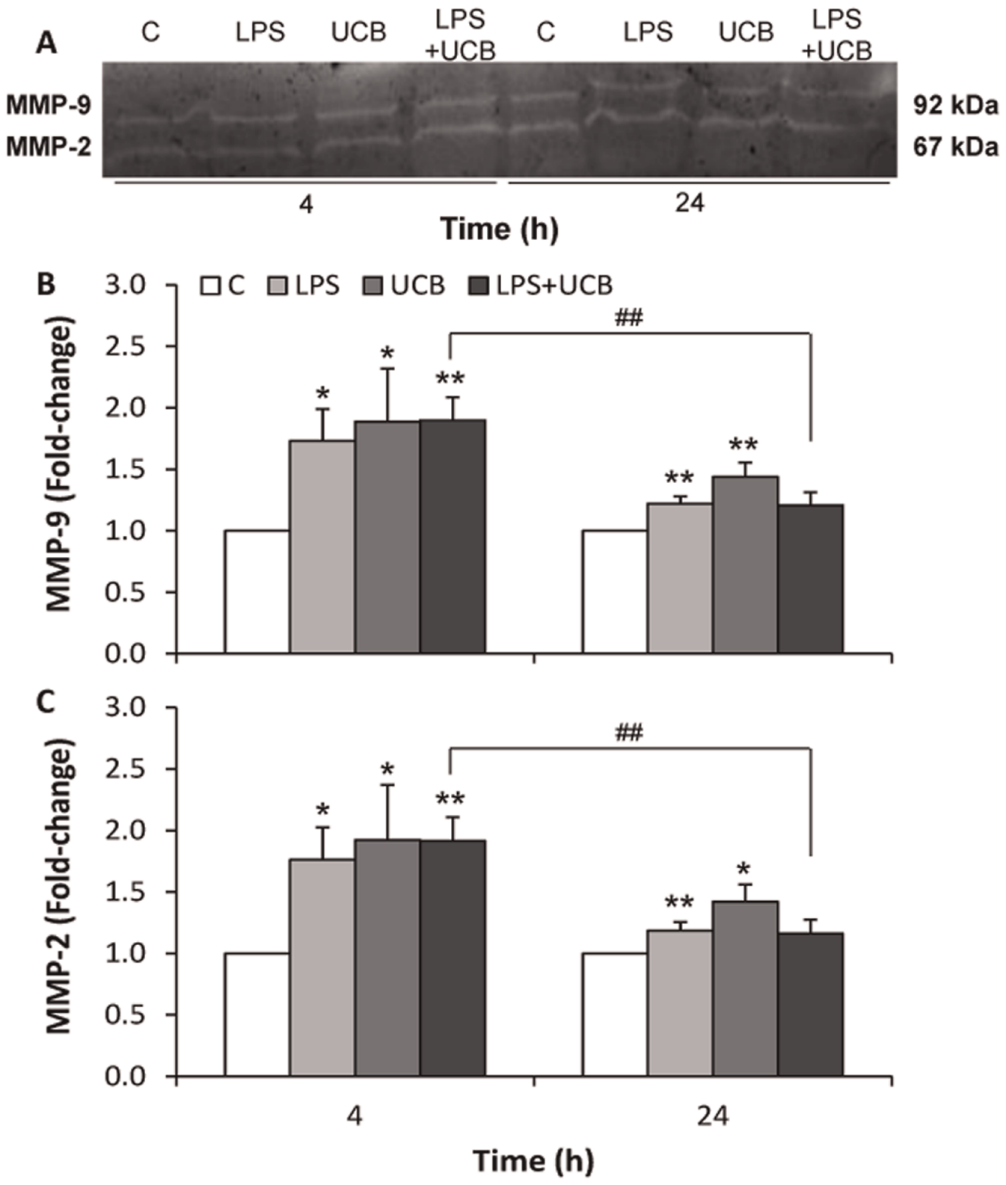
Lipopolysaccharide (LPS) and unconjugated bilirubin (UCB) activate metalloproteinase-9 (MMP-9) and metalloproteinase-2 (MMP-2) released by brain microvascular endothelial cells. A representative gel from one experiment is shown, where MMP-2 and MMP-9 were identified by their apparent molecular mass of 67 and 92 kDa, respectively (A). The intensity of the bands was quantified by scanning densitometry and results for MMP-9 (B) and MMP-2 (C) were standardized with respect to total protein content. Results are mean ± S.E.M. from at least four independent experiments performed in duplicate. *P<0.05 and **P<0.01 *vs.* respective control; ^##^ *P*<0.01 from 4 h.

It has been known that the release of active MMPs is related to the opening of the BBB in LPS-injured brain tissue [36]. The activities of MMP-2 and MMP-9 released by rat BMEC after exposure to the insults were similar (Fig. 3). Significant increases in the activity of secreted MMPs were already observed at 4 h for both LPS (1.7-fold for MMP-9 and 1.8-fold for MMP-2, *P<*0.05) and UCB (1.9-fold for MMP-9 and MMP-2, *P<*0.05), which were less marked at 24 h treatment though still statistically significant. No significant aggravation of the effect was observed by simultaneous exposure to both neurotoxins, either at 4 or at 24 h.

### LPS and UCB Alter Unique Ultrastructure of Rat BMEC

**Figure 4:**
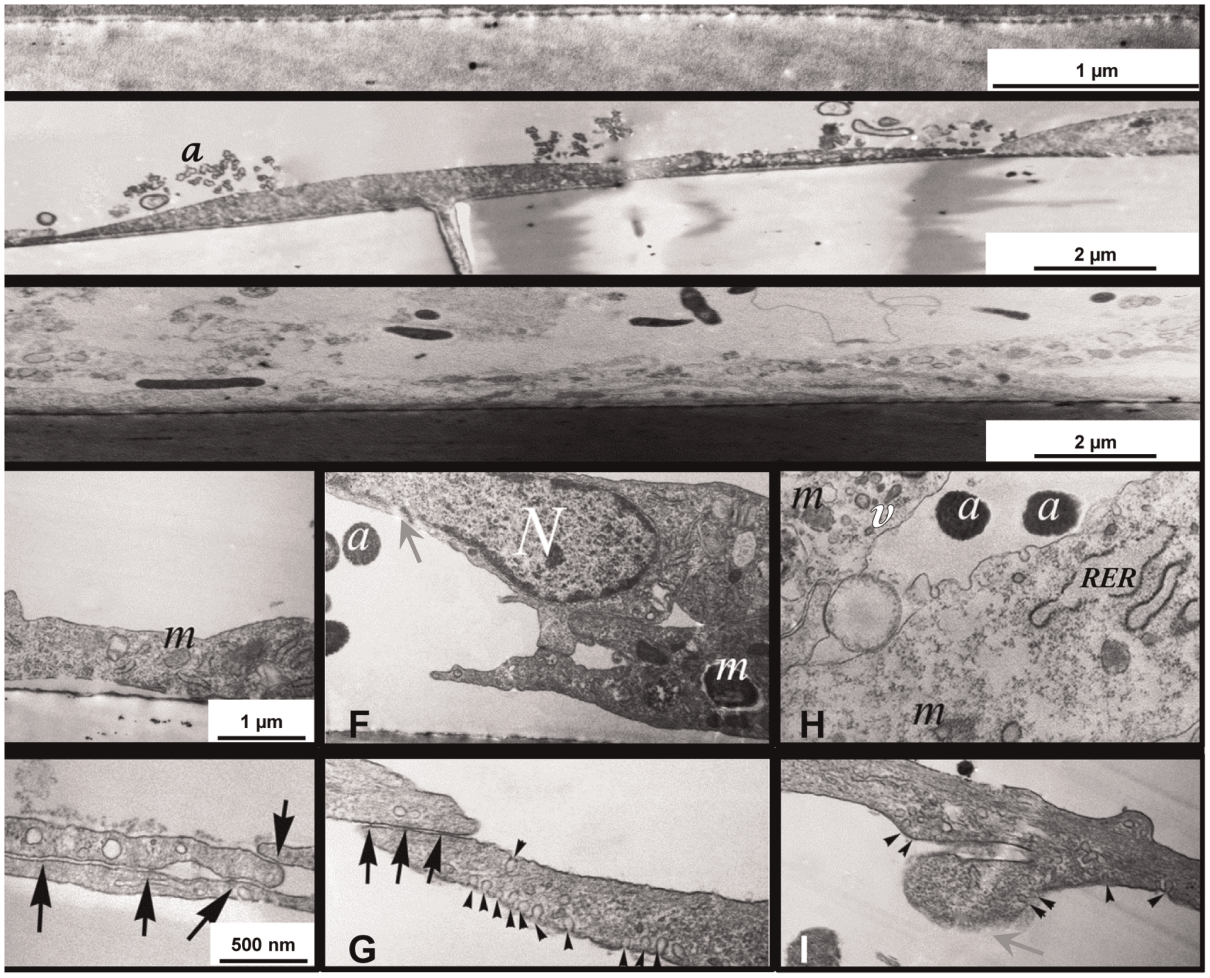
Lipopolysaccharide (LPS) and unconjugated bilirubin (UCB) disrupt ultrastructure of brain endothelial cells. Cells were treated with no addition (control) (A,D,E), LPS (B,F,G) or UCB (C,H,I) and were analyzed by transmission electron microscopy. Black arrows, tight intercellular junctions; grey arrows, disruption of the plasma membrane; arrowheads, invaginations of the plasma membrane; a, apoptotic cells bodies; m, mitochondria; N, cell nuclei; RER, rough endoplasmic reticulum; v, vacuole. Representative results from one of four independent experiments are shown.

Transmission electron microscopy (TEM) investigated in detail the impairment of rat BMEC monolayers exposed to the neurotoxins (Fig. 4). In cell cultures treated with LPS or UCB, several signs of cell damage and the presence of apoptotic bodies released by rat BMEC were detected, confirming the occurrence of apoptotic processes. Our observations include heavily damaged mitochondria that were swollen and more electron dense than in control cells, indicating an impairment in mitochondrial function in both conditions. In addition, the rough endoplasmic reticulum was typically swollen in the treated cells. This alteration may indicate a lesion of the synthesis of lipids or metabolism of sugars, and also impairment in their detoxification function. Moreover, it was observed irregularly shaped vacuoles and holes in the cytoplasm, together with unevenness and little projections instead of a rather smooth, elongated shape of the rat capillary endothelial cell. Appearance and abundance of caveolae-like invaginations in the plasma membrane was frequently found in LPS-treated cells, an effect that was not so evident in cells incubated with UCB. TEM analysis revealed shortened extension of intercellular contacts in LPS-treated cells and no intact cell-cell junctions could be detected in UCB-treated cells.

### Exposure to LPS and UCB Impair the Barrier Function of Rat BMEC

TEER and paracellular permeability to small molecule tracers provide information on barrier integrity in various *in vitro* BBB models [29], [33], [37]. We have demonstrated that LPS treatment caused a concentration- and time-dependent decrease in TEER and increased permeability to tracers in rat BMEC co-cultured with astrocytes [10], but, so far, such features have not been explored in cells exposed to UCB or to both neurotoxins, either in co-cultures or in BMEC mono-cultures.

**Figure 5:**
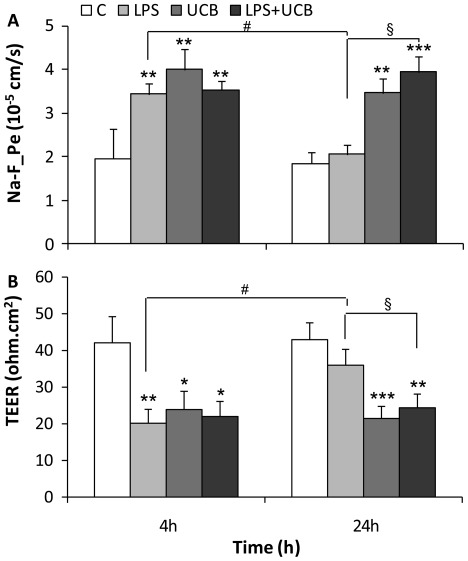
Lipopolysaccharide (LPS) and unconjugated bilirubin (UCB) disrupt the endothelial monolayer. Permeability to sodium fluorescein (Na-F_Pe) (A) and transendothelial electrical resistance (TEER) (B) were determined after exposure. All values presented are means ± S.E.M. from at least four independent experiments performed in duplicate. *P<0.05, **P<0.01 and ***P<0.001 *vs.* respective control; ^§^ *P<*0.05 *vs.* LPS at the same time-point;^ #^ *P*<0.05 from 4 h.

Permeability to sodium fluorescein (Na-F) was of ∼1.9×10^−5^ cm/s in control BMEC monolayers. LPS exposure induced an elevation of permeability to 3.4×10^−5^ cm/s at 4 h (*P<*0.01), and only a moderate increase after 24 h exposure (2.1×10^−5^ cm/s, *P*<0.05 *vs.* 4 h) (Fig. 5A). Similarly, cells treated with UCB showed an increased permeability at 4 h treatment (4.0×10^−5^ cm/s, *P<*0.01), with a sustained effect at 24 h. Alterations in permeability were significantly higher after simultaneous exposure to both insults at 24 h compared to those treated with LPS alone (*P<*0.05).

TEER was assessed after exposure to LPS and/or UCB for 4 h and 24 h (Fig. 5B), and results were compared with those obtained in controls (∼42 Ω cm^2^ after subtracting the values of cell free inserts). LPS treatment led to a significant decrease in TEER after 4 h exposure (20 Ω cm^2^, *P*<0.01) and monolayers partially recovered from the early onset damage at 24 h, as indicated by higher resistance levels (36 Ω cm^2^, *P*<0.05 *vs.* 4 h).

On the other hand, UCB caused lower values through longer periods of exposure, as indicated by the TEER decrease at 4 h (24 Ω cm^2^, *P*<0.05) and at 24 h (21 Ω cm^2^, *P*<0.001). Incubation with both LPS and UCB for 24 h did not aggravate UCB-induced effect, but TEER values were significantly lower than for 24 h treatment with LPS alone (*P<*0.01 as compared to the respective control, and *P<*0.05 from LPS alone).

**Figure 6:**
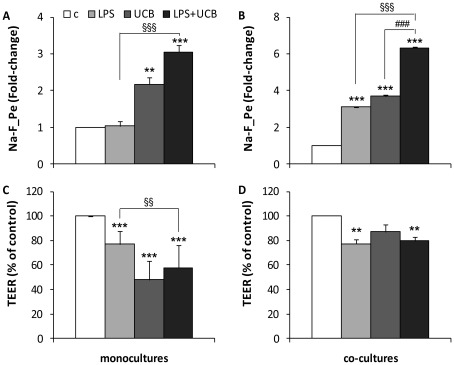
Effects of lipopolysaccharide (LPS) and unconjugated bilirubin (UCB) on endothelial integrity in mono-cultures and co-cultures. Permeability to sodium fluorescein (Na-F_Pe) (A, B) and transendothelial electrical resistance (TEER) (C, D) were determined in mono-cultures (A,C) and co-cultures (B,D) after 24 h exposure. All values presented are means ± S.E.M. from at least two independent experiments performed in triplicate. **P<0.01 and ***P<0.001 *vs.* respective control; ^§§^ *P<*0.01 and ^§§§^ *P<*0.001 *vs.* LPS at the same time-point.

Co-cultures of BMEC with astrocytes are accepted as more complex and tighter *in vitro* models of the BBB since astrocytes induce the barrier properties of BMEC [1], [29], [33]. To reinforce our observations, a set of experiments was performed in rat BMEC co-cultured with glial cells where the paracellular permeability to Na-F and the TEER were assessed 24 h after treatment (Fig. 6). In co-cultures, untreated BMEC had higher TEER (233 Ω cm^2^) and lower permeability (2.1×10^−6^ cm/s) values than mono-cultures. The permeability pattern was similar to that of mono-cultures, though in general 2-fold greater and there was a significant increase induced by LPS. Regarding TEER, the response to the neurotoxins was also similar in both models, but the alterations resulting from the presence of astrocytes were more modest as compared with mono-cultures, as well as in comparison with those observed for permeability, particularly in UCB-treated cells.

### Junctional Proteins in Rat BMEC are Altered by LPS and UCB

**Figure 7:**
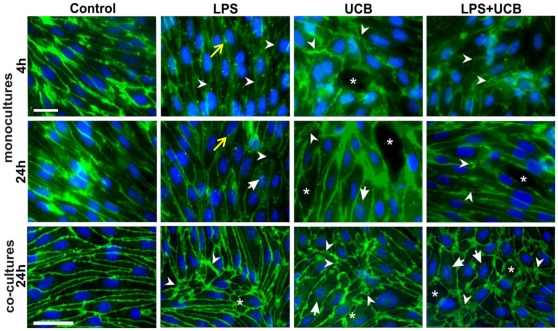
Lipopolysaccharide (LPS) and unconjugated bilirubin (UCB) modify the distribution of β-catenin in brain endothelial cells. Cells in mono-culture or co-cultured with astrocytes were fixed and immunostained with an antibody against β-catenin to evaluate its cellular localization (scale bars, 40 and 20 µm, respectively). Disruption of the monolayer with gaps between endothelial cells (*), alterations in protein patterns (arrowheads) with the presence of dot-like staining (yellow arrow), and perinuclear distribution (arrows) are indicated. Representative results from one of two independent experiments are shown.

Cellular expression and localization of the AJ protein β-catenin was also assessed in mono-cultures both at 4 h and at 24 h (Fig. 7). After LPS treatment cell contours were hardly visible at 4 h, whilst β-catenin was relocated at the membrane at 24 h. β-catenin appeared as small dots in LPS-treated cells, which were particularly evident at 4 h incubation, contrasting with the normal staining observed in control cells. Treatment with UCB also revealed alterations in the distribution of β-catenin, which varied with the time of exposure. In fact, a short incubation led to a transient translocation of the protein from the membrane to the cytosol; this effect appears to be transient as at 24 h the presence of the protein was clearly noticed at intercellular contacts. Interestingly, an overall increase in fluorescence intensity of β-catenin was detected in cells treated for 24 h with UCB (1.3-fold from control, *P*<0.01), whereas a decrease was observed for LPS (0.7-fold, *P*<0.01). As a result of these opposite variations, no significant change in the fluorescence intensity was observed in cells simultaneously treated with LPS and UCB.

Disorganization of the monolayer together with gaps between endothelial cells was also detected in cells exposed to the neurotoxins. In rat BMEC co-cultured with astrocytes not treated with LPS or UCB (control) for 24 h, β-catenin was mainly located at the plasma membrane. Compared to mono-cultures, LPS-treated cells also showed greater expression of β-catenin but with visible large intercellular spaces. In UCB-treated cells, on the other hand, the intensity of β-catenin immunostaining in the cytoplasm was weaker and that at the cell contours was greater than in mono-cultured cells at the same period. In BMEC treated with LPS and UCB, β-catenin was noticeable not only at the plasma membrane but also in the cytosol including the perinuclear region. Moreover, the staining revealed intercellular gaps.

**Figure 8:**
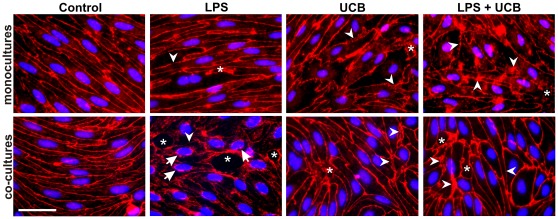
Lipopolysaccharide (LPS) and unconjugated bilirubin (UCB) alter *zonula occludens*-1 (ZO-1) expression in brain endothelial cells. Cells, either in mono-culture or co-cultured with astrocytes, were fixed and immunostained with an antibody against ZO-1 to evaluate its cellular localization and pattern of expression, as well as integrity of the monolayer. Disruption of the monolayer with gaps between endothelial cells (*), alterations in protein patterns (arrowheads) and perinuclear distribution (arrows) are indicated. Representative results from one of two independent experiments are shown. Scale bar, 20 µm.

**Figure 9:**
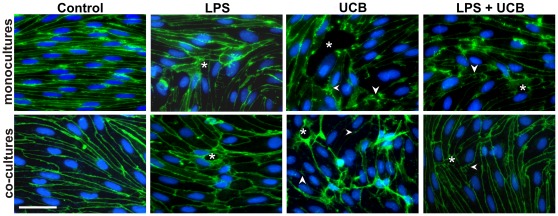
Lipopolysaccharide (LPS) and unconjugated bilirubin (UCB) alter expression of claudin-5 in brain endothelial cells. Cells, either in mono-culture or co-cultured with astrocytes, were fixed and immunostained with an antibody against claudin-5 to evaluate its cellular localization, pattern of expression and integrity of the monolayer. Disruption of the monolayer with gaps between endothelial cells (*) and alterations in protein patterns (arrowheads) are indicated. Representative results from one of two independent experiments are shown. Scale bar, 20 µm.

Immunostaining for TJ proteins ZO-1 and claudin-5 was performed in mono-cultures and co-cultures after 24 h of treatment with the neurotoxins (Figs. 8 and 9). In control cells, the contour line observed for ZO-1 appeared thicker in co-cultures than in mono-cultures, similarly to that observed for β-catenin. Despite the beautiful labeling obtained for claudin-5 in mono-culture control cells, the contours appeared lighter than in co-cultures. Moreover, the staining observed in the cytosol nearly disappeared in co-cultures and, therefore, the main immunostaining was at the plasma membrane when endothelial cells were co-cultured with astrocytes, for both ZO-1 and claudin-5. In mono-cultures, cells exposed to LPS or UCB alone, or to their combination, showed altered morphology and staining patterns for ZO-1 and claudin-5, as compared with untreated cells that showed elongated shape and well delineated contours. Cells treated with UCB, or its combination with LPS, showed fragmented staining for claudin-5, suggesting an impairment of the intercellular junctions. Moreover, spaces were visible between adjacent cells, further confirming the damaged connection between cells following treatment with the insults. Unlike the simplified model, co-cultured cells treated with LPS looked more altered at this time-point. They showed large spaces between cells and less staining for ZO-1 at the membrane, with more cytoplasmic and perinuclear immunoreactivity. On the other hand, UCB seemed to have a greater effect on claudin-5 with a decrease in plasma membrane immunoreactivity.

## Discussion

In the present study, confluent monolayers of primary rat BMEC were used alone or in co-culture as *in vitro* models of the BBB. The integrity of the endothelial barrier and its functional and morphological characteristics were evaluated after treatment with LPS and/or UCB. Our study shows that LDH release in primary rat BMEC is significantly modified by UCB and by both insults combined, but that at least 77% of cells are viable, which is a desirable experimental condition to perform functional studies such as the assessment of P-gp and MMPs activities. Necrotic-like cell death induced by the same concentration of LPS was reported in transformed bovine BMEC by Karahashi et al. [38], though the extent was over 40% at 24 h and no significant damage was seen at 4 h. Such divergent viability data may not only reflect different reactions to neurotoxins between species and between cell lines and primary cultures [39], but can mostly be due to the fact that this study was performed with medium supplemented with 10% FBS, unlike our own models. In studies performed in similar conditions to ours, namely with no addition of soluble CD14, there is also little LPS-induced LDH release for up to 16 h, being this the maximum time studied [34]. Still, our conditions are suitable for LPS interaction with BMEC given that our medium contains 2% FBS, which has soluble CD14, and that EC also express membrane CD14 in the presence of LPS [8]. LDH release induced by UCB is in agreement with our previous observations in a cell line of human BMEC [27], although in a slightly different range. Apoptotic cell death resulting from exposure to LPS is observed in rat primary BMEC, and the present data are in agreement with that obtained in cell lines of rat, bovine and human BMEC [38], [40]. UCB-induced apoptosis, based on analysis of nuclear morphology with Hoechst staining and confirmed by caspase-3 activity, is in line with our previous observations in a human BMEC line treated with UCB [27], as well as with those of Akin et al. [41] in bovine BMEC. An interesting observation from this study is that the cell damage shown by LDH release, apoptotic cell death and caspase-3 activation tend to be higher and occur earlier in UCB-treated cells than in LPS-exposed ones. Accordingly, LPS did not further enhance UCB-induced cell demise. On the other hand, while no significant apoptosis or caspase-3 activation were detected by LPS alone at 4 h, cells co-exposed to both insults at this time-point exhibited a similar profile to that of UCB.

P-gp is an important efflux transporter at the BBB, which protects the nervous system from toxic compounds. Therefore, its inhibition compromises brain protection and promotes toxicity. The retention of R123 by LPS in our experiments reflects an impairment of the transporter activity and confirms previous reports obtained both *in vitro* [10], [42] and *in vivo* [43], [44]. The data collected also revealed an inhibition of P-gp activity by UCB, which is a substrate of P-gp in bovine BMEC [45]. Brain UCB levels were increased in P-gp deficient mice [46] and in rats treated by P-gp inhibitors [46], [47]. It is interesting to point out that ongoing studies in our laboratory revealed higher expression of P-gp in the brain parenchyma (endothelial cells, astrocytes and neurons) of a human newborn infant who died from kernicterus, the most severe pathologic outcome of hyperbilirubinemia (unpublished data). These results suggest that the increase in P-gp expression may be a compensatory mechanism to overcome the lack of efficiency in its activity at the BBB. On the other hand, if UCB levels inside endothelial cells are extremely elevated, the transporter might not have the capacity to export both UCB and R123 simultaneously, resulting in higher R123 contents.

MMPs are a family of proteases, important in normal development and in endothelial cell migration given their ability to degrade the ECM surrounding BBB capillaries [48]. As ECM plays an important role in the induction of BBB properties [1], the activity pattern of secreted MMPs after exposure to LPS and/or UCB was investigated in the present study. Both MMP-2 and MMP-9 have similar activity patterns, which may be due to a homologous function. Our data are in agreement with previous studies where it has been proven that exposure to LPS results in enhanced secretion of active MMP-2 in human umbilical vascular endothelial cells [49], and that microglial cells treated by UCB secrete active MMP-2 and MMP-9 [26]. Released MMPs may lead to degradation of the ECM and weaken the barrier properties of the BBB by impairing the integrity of the basement membrane [50], which might then favor the access of neurotoxins to the brain. Interestingly, activation of MMPs is more marked at 4 h than at 24 h, consistent with the striking alterations observed at earlier time for permeability and TEER.

Ultrastructural analysis has indicated that apoptotic cell bodies are visible following both LPS and UCB treatments, supporting our apoptosis data obtained by fluorescence microscopy. All treated cells presented disruption of the organelles, but it was interesting to notice that the impairment of ultrastructure was more marked for UCB than for LPS. This observation is consistent with the greater extent of apoptotic and necrotic-like cell death by UCB and might be related to UCB interaction with cell membranes and perturbation of their dynamic properties that we have previously demonstrated [51], [52]. Another important observation is the enhanced formation of caveolae-like structures after LPS exposure, in line with previous observations in rats [53], [54]. Caveolin-1 plays an important role in capillary formation and, when up-regulated, it activates MMP-2 and MMP-9 [49], [55]. These MMPs were particularly amplified after exposure to LPS for 4 h, thus pointing to a possible relation between these partners.

Rat BMEC exposed to LPS still present TJs, though the TJ-stiched membrane length appeared shorter compared to untreated samples, whereas TJs are hardly visible in UCB-treated cells. This observation may suggest that paracellular passage of UCB across the endothelial monolayer may be facilitated. Although a TEM study by Böhm et al. [56] reported UCB-induced damage of both cell and nuclear membranes in human basal skin cells, as far as we know, this is the first report describing UCB-induced ultrastructural alterations in endothelial cells forming the BBB.

Our permeability results on brain endothelial mono-cultures are in line with those recently obtained by He and collaborators [57], where a short exposure to LPS induced hyperpermeability and TEER decrease in a BMEC line. Moreover, a study reports that TLR4 and CD14 mRNA levels increase up to 3 h after LPS exposure and then gradually decrease, though remaining detectable for up to 9 h [8]. This observation might explain why LPS effects in mono-cultures become less marked as the incubation is prolonged. This fact, together with the higher activity of MMPs by a short exposure, shall then contribute to the greatest impairment of barrier indicators observed at 4 h. In co-cultures, the present results are supported by our previous reports that LPS elevates permeability and decreases TEER [10]. It has been long stated that astroglial cells strengthen BMEC barrier properties [1], [29], [33] and prevent the increase of endothelial permeability after several stimuli, such as hypoxia or ischemia [58], [59]. The protective effect of glial cells against LPS-mediated injury was also demonstrated in bovine brain endothelial cells [60]. Nonetheless, it has been proven that, in inflammatory conditions, astrocytes increase human BMEC permeability via IL-1β secreted to the cell culture supernatant and this could be reversed through a monoclonal antibody against IL-1β [61]. Regarding UCB, no former studies have been performed on its effects on brain endothelial barrier properties. Raimondi et al. [62] demonstrated that exposure of human intestinal cell line Caco-2 to UCB decreases transepithelial electrical resistance and increases the paracellular flux of 10 kDa dextran. Our present findings obtained in rat BMEC regarding UCB-induced decrease in TEER and increase in permeability are consistent with the data found in Caco-2 epithelial cells [62]. Unlike the intestinal cells, rat BMEC monolayers do not recover at later time-points, indicating high endothelial sensitivity to UCB toxicity and a greater potential of UCB for neurotoxicity. Endothelial cells treated with both compounds for 24 h displayed more serious barrier dysfunction as compared with LPS alone, pointing to a failure of the recovery process that might happen when sepsis occurs simultaneously with hyperbilirubinemia. IL-6 is released by human BMEC, which additionally produce nitric oxide [27], a molecule that contributes to enhance the permeability of the barrier [1]. Rat BMEC produce prostacyclin, as we have previously demonstrated [63]. Thus, changes in prostanoid, especially prostacyclin synthesis [64] may also contribute to the observed endothelial permeability alterations. Interestingly, an increased permeability and a decreased TEER were also observed when each of the neurotoxins, or their combination, were added to BMEC in co-culture with astrocytes. This observation, relying in an *in vitro* model that more closely resembles the *in vivo* condition, reinforces the data obtained in the simpler model of the BBB composed of BMEC alone.

Analysis of the AJ protein distribution pattern revealed a decreased localization at intercellular junctions at 4 h exposure to LPS, UCB or their combination, which is partially reversed at 24 h, as well as a disorganization of the endothelial sheet, particularly evident in co-cultured cells. Also interesting was the perinuclear accumulation of β-catenin, which was associated with a disruption of the VE-cadherin/β-catenin complexes during inflammation [65], [66]. Therefore, the production of cytokines by astrocytes and BMEC exposed to LPS or UCB [27], [32], [67], [68] may contribute to this process, which brings new insights into the mechanisms of toxicity by these compounds. Since Wnt signaling pathway activation is recognized as a mechanism of cell survival involved in the expression of TJ-associated proteins [69], the present observations suggest an attempt to compensate the loss of integrity resulting from exposure to the insults.

The expression and localization of the TJ proteins ZO-1 and claudin-5 are also affected by the tested neurotoxins. The accumulation of these proteins in the plasma membrane of co-cultured control cells, compared to mono-cultured cells is in line with the astrocytic induction of barrier properties in endothelial cells *in vitro* [1], [29], [33]. It may also be related with the low serum concentration used in this study, as astrocyte co-cultures in the absence of serum in the basolateral compartment result in higher BMEC barrier indices, given their greater resemblance to the *in vivo* microenvironment [70]. Our mono-culture results confirm that LPS treatment alters intercellular junctions that are associated with BBB impairment [71]. UCB-treated cells also show a redistribution of TJs proteins similarly to what was seen for occludin in epithelial cells [62]. The segmentation of claudin-5 immunostaining pattern may be related to the secretion of active MMPs, as it was described for occludin [72], [73]. Considering that Yang et al. [74] have reported a reduction of claudin-5 mRNA in mice following an increase in MMP-2 expression, it is conceivable that activation of MMPs might be related to the loss of claudin-5. To the impairment of TJs and consequent increase in permeability may also account nitric oxide [75], which is produced by exposure of BMEC and glial cells to UCB or LPS [10], [27], [76].

The increase in the permeability of co-cultured rat BMEC exposed to the neurotoxins might be related to the alterations in the pattern of expression and cellular localization of ZO-1 and claudin-5, already demonstrated at earlier time-points [10]. In fact, the mRNA contents of TJ proteins ZO-1 and claudin-5, as well as their cellular localization decrease in bovine retinal endothelial cells treated by IL-1β and TNF-α [77], which are known to be secreted by astrocytes. The translocation of ZO-1 from the membrane to the nucleus has never been reported in endothelial cells treated with LPS. Still, a recent paper by Zhong and collaborators [78] describes that ZO-1 is present in small concentrations in the nuclei, even in untreated cells. Therefore, our results are in agreement with the dual role of ZO proteins [79], and the evident increase of the perinuclear staining brings new insights into a possible translocation of ZO-1 to the nucleus or acting as a nuclear factor during inflammation in the presence of astrocytes. Its dissociation from the junctional complexes is related with increased barrier permeability [80], which may further explain the mechanism for enhanced permeability in co-cultures. The same permeability enhancement in UCB-treated cells can also be related to the redistribution of claudin-5, an important contributor to TJs seal [1].

### Conclusions

The present data prove that LPS and UCB alter various aspects in rat BMEC. We can conclude that these neurotoxins diminish the viability of brain endothelial cells and alter BBB functions by inhibiting P-gp activity and inducing the secretion of active MMPs. Ultrastructural analysis detected newly formed caveolae-like structures and damage of organelles, like mitochondria and rough endoplasmic reticulum, which may be involved in the mechanisms of action of these neurotoxins. Moreover, this is the first study to demonstrate that paracellular permeability, TEER and intercellular junctions are altered by these compounds in rat BMEC either in mono-cultures or in co-culture with astrocytes.

At last, our study revealed that each of the compounds has its own mechanism of action and temporal profile, comprising cell death (early occurring for UCB and only lately for LPS), the impairment of barrier properties (sustained along time for UCB but not for LPS), the distribution pattern of intercellular junctions as β-catenin (opposite variations in fluorescence intensity for UCB and LPS) and ultrastructural alterations (shortened intercellular junctions and the increased number of caveolae particularly evident in LPS-treated cells). Collectively, by compromising the BBB integrity, the alterations resulting from exposure to LPS and/or UCB may facilitate the access of the compounds to the brain further contributing to their neurotoxicity.

## Materials and Methods

### Cell Cultures

Animal care followed the recommendations of European Convention for the Protection of Vertebrate Animals Used for Experimental and other Scientific Purposes (Council Directive 86/609/EEC) and National Law 1005/92 (rules for protection of experimental animals). Formal approvals to conduct all animal procedures in the experiments have been obtained from the Animal Experimentation Committee of the Biological Research Centre, Hungarian Academy of Sciences (Hungary), and from the local authorities (Permit number: XVI./03835/001/2006).

Primary cultures of rat BMEC were prepared from 2-week-old Wistar rats, as previously described [10], [81], [82]. Briefly, meninges were removed from forebrains and gray matter was minced into small pieces in Dulbecco’s modified Eagle’s medium (DMEM)/Ham’s F12 (DMEM/F12, Biochrom AG, Germany), and digested in DMEM/F12 containing 1 mg/ml collagenase CLS2 (Worthington, USA) for 1.5 h at 37°C. Cell pellet was separated by centrifugation in 20% bovine serum albumin-DMEM/F12 (1000×*g*, 20 min). Microvessels were further digested with 1 mg/ml collagenase-dispase (Roche Applied Sciences, Switzerland) in DMEM/F12 for 1 h at 37°C. Microvessel endothelial cell clusters were separated on a 33% continuous Percoll (Pharmacia, Sweden) gradient, collected and washed in DMEM/F12 before plating (1.5×10^5^ cells/cm^2^) onto collagen type IV and fibronectin coated cell culture inserts or multiwell plates. Cultures were maintained in DMEM/F12 supplemented with 15% fetal bovine serum (FBS) (Biochrom AG), 1 ng/ml basic fibroblast growth factor (Roche Applied Sciences), 100 µg/ml heparin (Biochrom AG), 5 µg/ml gentamycin and 4 µg/ml puromycin [81] (medium I) at 37°C with a humidified atmosphere of 5% CO_2_, for 2 days. On day 3, cells received new medium, which contained all components of medium I except puromycin (medium II).

Primary cultures of glial cells were prepared from newborn Wistar rats [10], [81], [82]. Meninges were removed, and cortical pieces were mechanically dissociated in DMEM containing 5 mg/ml gentamicin and 10% fetal bovine serum and plated in poly-l-lysin coated 12-well dishes and kept for minimum 3 weeks before use. In confluent astroglia cultures 90% of cells were immunopositive for the astroglia cell marker glial fibrillary acidic protein, while the remaining 10% was immunopositive for CD11b, a marker of microglia.

For co-culture, BMEC in cell culture inserts were placed into multiwells containing glia at the bottom of the wells with endothelial culture medium in both compartments. When BMEC became almost confluent 550 nM hydrocortisone was added to the culture medium [83], for 1 day.

### Treatment of Endothelial Cells

UCB (Sigma-Aldrich, USA), was purified according to the method of McDonagh and Assisi [84] and stock solutions were extemporarily prepared in 0.1 M NaOH, under light protection, and the pH adjusted to 7.4 by addition of equal amounts of 0.1 M HCl. A stock solution of LPS (*Escherichia coli* O111:B4; Calbiochem, Germany) was prepared in PBS. Confluent monolayers of rat BMEC received medium II with 2% FBS, and were divided into four groups: untreated cells (control), 1 µg/ml LPS, 50 µM UCB, in the presence of 100 µM human serum albumin (fraction V, fatty acid free, Sigma-Aldrich), or a combination of both, for 4 h and 24 h at 37°C. The concentration of LPS was selected to mimic sepsis [85]–[87] and was shown to induce damage to rat BMEC in our previous study [10]. The use of albumin mimics the *in vivo* condition, where there is equilibrium between the bilirubin fraction that is bound to albumin, the one that is free in circulation and the one that binds to cells. Therefore, albumin functions as a sink for bilirubin, so that bilirubin may be continuously released from albumin as it binds to cells, therefore perpetuating the fraction that is available for interaction with cells and thus enhancing the effects. The UCB condition mimics moderate to severe hyperbilirubinemia in a term or a preterm infant, respectively [88]. In fact, studies performed in our lab showed that a group of full term neonates with moderate jaundice present a molar ratio of 0.42 (∼195 µM bilirubin and ∼445 µM serum albumin) [89], and that a preterm infant with a bilirubin to albumin molar ratio of 1.0 (∼493 µM bilirubin and ∼498 µM serum albumin) at the second day of life dyed on the fourth with the diagnosis of kernicterus [90]. In the present experimental conditions, the concentration of unbound UCB (free UCB), determined by the peroxidase method [91], was 12 nM, which is close to the 19 nM value found in the moderate jaundiced infants studied [89]. The absence of biliverdin in the UCB-treated samples was assured by continuous absorbance spectra from 300 to 800 nm [92], which revealed no peaks at 380 and 665 nm.

### Cell Death Evaluation

Standard evaluation of rat BMEC cytotoxicity was performed by measuring the release of LDH from cells with damaged plasma membrane into the incubation medium using a cytotoxicity detection kit (Roche Molecular Biochemicals, Germany), as previously described [39]. The reaction was performed in a 96-well microplate and the absorbance measured at 490 nm, using a PR 2100 microplate reader from Bio-Rad (USA). All readings were corrected for possible interference of UCB absorption and the results expressed as percent of LDH release. Cytotoxicity was calculated as percentage of the total LDH release from untreated cells lysed with 2% Triton X-100 for 30 min. To evaluate apoptosis, cells fixed in freshly prepared 4% paraformaldehyde solution in phosphate buffer saline (PBS) were immunostained with Hoechst 33258 dye for 2 min at room temperature, and mounted in Glycerol Mount. Apoptotic nuclei were identified by condensed chromatin or nuclear fragmentation, and counted for each independent experiment in at least five random microscopic fields (400×) per sample, as described previously [32], using a Leica DFC 490 camera (Leica, Germany) adapted to an AxioScope.A1 microscope (Zeiss, Germany). Although the fluorescence emission of Hoescht 33258 dye at 461 nm overlaps with the absorption of UCB at 460 nm, the possible quenching by UCB absorption does not interfere with the obtained results since apoptosis was evaluated based on morphology and not on fluorescence intensity. Activity of caspase-3 was measured by a colorimetric method (Calbiochem, Darmstadt, Germany), as usual in our lab [27]. Briefly, cells were harvested, washed with ice-cold PBS, and lysed for 30 min, on ice, in the lysis buffer [50 mM 4-(2-hydroxyethyl)-1-piperazineethanesulfonic acid (pH 7.4); 100 mMNaCl; 0.1% (w/v) cholamidopropyldimethylammonio-1-propanesulfonate; 1 mM dithiothreitol; 0.1 mM ethylenediaminetetraacetic acid]. The lysate was centrifuged at 10,000×*g* for 10 min at 4°C and the supernatants were collected and stored at −80°C. The activity of caspase-3 was determined by enzymatic cleavage of chromophorep NA from the substrate, according to manufacturer’s instructions. The proteolytic reaction was carried out in protease assay buffer [50 mM 4-(2-hydroxyethyl)-1-piperazineethanesulfonic acid (pH 7.4); 100 mM NaCl; 0.1% (w/v) cholamidopropyldimethylammonio-1-propanesulfonate; 10 mM dithiothreitol; 0.1 mM ethylenediaminetetraacetic acid; 10% (v/v) glycerol], containing 2 mM substrate Ac-DEVD-pNA. Following incubation of the reaction mixtures for 2 h at 37°C, the formation of pNA was measured at 405 nm with a reference filter of 620 nm. The results were expressed as relative activity *vs*. control samples.

### Functional Assay for P-gp

Activity of P-gp was determined by measuring cellular accumulation of the P-gp substrate R123 per mg of protein content [10], and results were expressed as fold-change as compared to the respective control. In brief, treated rat BMEC were washed, and incubated for 1 h at 37°C with Ringer–Hepes solution (118 mM NaCl, 4.8 mM KCl, 2.5 mM CaCl_2_, 1.2 mM MgSO_4_, 5.5 mM d-glucose, 20 mM Hepes, pH 7.4) containing 10 µM R123. The solution was quickly removed, rat BMEC were washed three times with PBS and solubilized in 0.1 M NaOH. R123 content was determined using a Polarstar Galaxy fluorescent plate reader (BMG Labtechnologies; excitation at 505 nm, emission at 538 nm). The fluorescence emission of R123 is not quenched by bilirubin’s absorption, as the absorption of UCB is at 460 nm. Verapamil (100 µM, 30 min pre-incubation) was used as a reference P-gp inhibitor for the positive control (1.9-fold at 4 h and 2.8-fold at 24 h). Protein content was evaluated by the Bradford method [93] using Bio-Rad’s Protein Assay reagent (Bio-Rad).

### Gelatin Zymography

Determination of MMP-2 and MMP-9 was evaluated as previously described [26]. In short, aliquots of culture supernatants were analyzed by sodium dodecyl sulfate polyacrylamide gel electrophoresis (SDS-PAGE) zymography in 0.1% gelatine–10% acrylamide gels under non-reducing conditions. After electrophoresis, gels were washed for 1 h with 2.5% Triton X-100 (in 50 mMTris pH 7.4, 5 mM CaCl_2_, 1 µM ZnCl_2_) to remove SDS and renature the MMPs species in the gel. Then the gels were incubated in the developing buffer (50 mM Tris pH 7.4, 5 mM CaCl_2_, 1 µM ZnCl_2_) overnight to induce gelatin lysis. For enzyme activity analysis, the gels were stained with 0.5% Coomassie Brilliant Blue R-250 (Bio-Rad) and destained in 30% ethanol/10% acetic acid/H_2_O. Gelatinase activity, detected as a white band on a blue background, was quantified by computerized image analysis and normalized with total cellular protein using Quantity One 1-D Analysis Software (Bio-Rad).

### TEM Analysis

Rat BMEC mono-cultures grown on inserts were fixed with freshly prepared 4% paraformaldehyde in 0.05 M cacodylate buffer (pH 7.5) for 30 min at 4°C. After washing with cacodylate buffer several times, the membranes of the cell culture inserts with the cells were removed from their support and placed into 24-well chamber slide and were post-fixed in 1% OsO_4_ for 30 min. Following washing with distilled water, the cells on the membrane were dehydrated in graded ethanol, block-stained with 1% uranyl acetate in 50% ethanol for 30 min and embedded in Taab 812 (Taab; Aldermaston, UK). Following polymerization at 60°C for 12 h, 50–60 nm ultrathin sections were cut perpendicularly for the membrane using a Leica UCT ultramicrotome (Leica Microsystems, UK) and examined using a Hitachi 7100 transmission electron microscope (Hitachi Ltd., Japan). Electron micrographs were made by Veleta, a 2 k×2 k MegaPixel side-mounted TEM CCD camera (Olympus). Electron micrographs were edited by Adobe Photoshop CS3 (Adobe Photoshop Incorporation, CA, USA).

### Evaluation of the Monolayer Integrity

The flux of Na-F (MW: 376 Da) across endothelial monolayers and co-cultures was determined as previously described [10], [82]. Briefly, cell culture inserts were transferred to 12-well plates containing 1.5 ml Ringer–Hepes solution in the abluminal compartments. Culture medium from luminal compartments was replaced by 0.5 ml Ringer–Hepes solution containing 10 µg/ml Na-F. Inserts were transferred at 20, 40 and 60 min to new wells containing Ringer–Hepes solution. Na-F levels were measured using a Hitachi F-2000 fluorescence spectrophotometer (excitation: 440 nm, emission: 525 nm). Flux across cell-free inserts was also measured. Endothelial permeability coefficient was calculated as previously described [10], [29] and results were expressed in cm/s or as fold-change compared to the respective control. TEER of mono-cultures and co-cultures, reflecting the paracellular permeability for mainly sodium ions in the present culture conditions, was measured using a STX-2 electrode coupled to an EVOM resistance meter (World Precision Instruments, USA). TEER readings of cell-free inserts (∼100 Ω cm^2^) were subtracted from the values obtained with cells. For comparison of the data between mono-cultures and co-cultures TEER of cells treated with no addition (control) was considered 100%.

### Immunostaining for Intercellular Junctions

BMEC in mono-culture or co-cultured with astrocytes were stained for junctional proteins ZO-1, claudin-5 and β-catenin. Cultures were washed in PBS and fixed with ethanol (95%)–acetic acid (5%) (v/v) for 10 min at −20°C [10]. Cells were blocked with 3% BSA and incubated with primary antibodies anti-ZO-1 (1∶200), anti-claudin-5 (1∶200), and anti-β-catenin (1∶200) (Invitrogen, USA) overnight at 4°C. Incubation with secondary antibody Cy3-labelled anti-rabbit IgG (1∶200) (GE Healthcare, UK) and Alexa 488 anti-mouse IgG (1∶500) (Invitrogen) lasted for 1 h at room temperature. Nuclei were counterstained with Hoechst 33258 dye. Between incubations cells were washed three times with PBS. Coverslips were mounted in Gel Mount (Biomeda, USA) and staining was examined by a Nikon Eclipse TE2000 fluorescent microscope (Nikon, Japan) and photographed using a Spot RT digital camera (Diagnostic Instruments, USA) or using a Leica DFC 490 camera (Leica, Germany) adapted to an AxioScope.A1 microscope (Zeiss, Germany). Evaluation of fluorescence intensity of β-catenin in mono-cultures was determined using ImageJ 1.29× software (N.I.H., USA) and results were expressed as fold change (mean fluorescence per number of cells *vs.* control samples).

### Statistical Analysis

Results of at least three different experiments were expressed as mean ± S.E.M. Differences between groups were determined by one-way ANOVA using Prism 5.0 (GraphPad Software, San Diego, CA) followed by multiple comparisons Bonferronipost-hoc correction. Statistical significance was considered when *P* values were lower than 0.05.
